# Explicating How Skill Determines the Qualities of User-Avatar Bonds

**DOI:** 10.3389/fpsyg.2022.713678

**Published:** 2022-03-22

**Authors:** Teresa Lynch, Nicholas L. Matthews, Michael Gilbert, Stacey Jones, Nina Freiberger

**Affiliations:** School of Communication, The Ohio State University, Columbus, OH, United States

**Keywords:** avatars, information processing, skill, affordances, action coupling, embeddedness, video games, motivation

## Abstract

Many frameworks exist that explain how people interact with avatars. Our core argument is that the primary theoretical mechanisms of a user-avatar bond (i.e., UAB) rest with the way people engage avatars and, thereby, the broader digital environment. To understand and predict such engagement, we identify a person’s skill in handling/engaging the avatar in the digital environment as an ordering parameter (i.e., organizing predictor). Accordingly, we define skill as a person’s ability to enact their agency successfully to achieve desired states. To explain how skill orders experience, we ground our theorizing in ecological perception and systems theory. In our explication, we describe how stable action coupling (i.e., the linking of action inputs to perceived outcomes) enables a state of embeddedness (i.e., when the environment facilitates and constrains behaviors) in the digital environment. Then, we explain how embeddedness promotes motivational attunement (i.e., orienting of motivational systems) and what the digital environment affords to users at different levels of skill. Throughout, we consider how our theoretical scaffolding generates tractable contentions regarding how skill influences UABs.

## Introduction

User-avatar bonds (UABs) are relational bonds that emerge from experiences a user has with an avatar in a digital environment. Definitions relevant to the broader construct of UABs abound in the literature (see Nowak and Fox, 2018 for a relevant review). As with any other relationship, a UAB develops over time through experiences the user has with the avatar. Similar to interpersonal relationships, users may experience stages in bonding with their avatar characterized by initiating contact and integrating identities (Knapp, 1978). Similar to parasocial relationships established with others in one-way digitally mediated contexts (Horton and Wohl, 1956), users may experience feelings of intimacy and closeness to their avatars. However, the relationship formed between a user and an avatar is distinct from interpersonal and parasocial relationships because users fundamentally transmit their agency through avatars. The user objectifies the avatar by manipulating it in ways that vary depending on its construction and presentation. The immediate and persistent thoughts, feelings, and behaviors the user has during and following interaction with the avatar in a digital environment characterize the nature of the bond. Accordingly, the UAB can take many qualitatively distinct forms as captured by scholars who have considered avatar relations (e.g., Banks, 2015).

In this writing, we define the *avatar* as the representation of the user’s agency in a digital environment. We define the *digital environment* as a system comprised of information transmitted digitally to the user *via* the hardware and software that hosts the avatar. We exclude the avatar from this definition to preserve its conceptual distinctiveness. Social scientists studying video games and other digital environments have long focused research on the interactions of a user (e.g., a video game player) and an avatar (e.g., the player’s character). Accordingly, many frameworks exist seeking to articulate specific phenomena that occur during or following these interactions. For example, identification (e.g., Klimmt et al., 2009) explains the adoption of an avatar’s characteristics into one’s own self-perception. Player-avatar relationships (PAR; Banks, 2015) describe how interactions with avatars can produce connections with avatars that vary in characterization (e.g., as useful tools, psychologically merged symbiotes, and distinct social others). And, the Proteus effect (Yee and Bailenson, 2007) demonstrates that characteristics of avatars’ representations can influence users’ social behaviors predictably.

In this work we centralize a person’s skill in handling or engaging the avatar in the digital environment. The argument we make in this paper is that the primary theoretical mechanisms of a UAB rest with the way the person engages the avatar and, thereby, the broader digital environment. Consequently, the full scope of UABs is not available to all players by default. Instead, skill-based prerequisites limit the range of possible experiences and, therefore, qualities of the relationship (e.g., a person must have some ability to control an avatar to feel competent). Despite this focus on characteristics of the person, we acknowledge that characteristics of the avatar and the digital environment influence the likelihood and nature of these states in important ways (e.g., both the avatar and the environment can vary in terms of form and function). Yet, as we will illustrate, even in similar digital environments with similar avatars, a person’s skill can differentiate the action patterns that form the basis of experiences. Because these experiences build to form UABs, skill differentiates UABs.

In this writing, we focus our theorizing on the highly interactive and complex digital environments of video games to demonstrate the applicability of our contentions. A number of scholars have promoted the notion that video game contexts are unique. Thus, we end by speculating on how the characteristics of video games and other highly interactive media experiences may produce unique relational bonds in addition to those already identified in the literature (e.g., monadic identification).

## Skill Differentiates Experiences With Avatars in Digital Environments

In the context of video games, *skill* is a player’s ability to successfully enact their agency in the digital environment. Skill varies between users with some individuals having relatively lower levels (i.e., novice) to higher levels (i.e., expert) than others. Users also, however, have varying levels of skill at different types of games. For example, Binta may have a high level of skill in a turn-based role-playing game, but she may have a low level of skill in a first-person shooter. Similarly, Binta may have had a high level of skill in a game at a point in the past that she has not maintained and, thus, her skill may have diminished over time. To meaningfully experience interactions that form the basis of a user-avatar bond, users must attend to and, to some degree, process information from those experiences. The variance that exists in a user’s skill produces variance in the nature of the experiences that a user will have with their avatar and their memory for those experiences.

In many video games, avatars translate a user’s commands from the input source (e.g., pressing the A-button on a controller to jump) into feedback the user can perceive in the digital environment (e.g., the avatar performing a jumping animation). Although certain conventions (e.g., control schemes, genre, etc.) allow skill to translate from game to game, it is a *situated* concept, meaning that its manifestation depends on a specific environmental context of task-relevant inputs and outputs (Wilson, 2002). For example, a user’s skill may take the form of a high kill-death ratio in a first-person shooter game, a short completion time (i.e., speed run) in a platformer game, complete exploration of a large map in a survival adventure game, or establishing and leading a guild in a massive multiplayer online game. Therefore, skill does not necessarily determine the likelihood of a certain outcome (e.g., winning, goal achievement). Rather, skill determines both *what* a digital environment affords the player and the degree to which a user can enact their agency effectively.

### Action Coupling as a Key Component of Skill

Successfully enacting one’s agency in the digital environment through an input source (e.g., a controller) requires that the user develop a proficiency at inputting commands to produce desired outcomes in the digital environment. More simply, this process requires the establishment of cognitive associations between inputs with the outcomes of those actions (i.e., outputs). Action coupling is one way to conceptualize such input-output associations. *Action coupling* is the associative linking of an action to its perceptual effect or outcome (see Novembre and Keller, 2014 for review). Action coupling has been studied in a variety of contexts (e.g., the coupling of instrumentalists’ movements and the musical sounds produced). Behavioral and neurophysiological studies of action coupling indicate that it functions to support prediction of outcomes (e.g., action planning; Pfordresher, 2005) and behavioral adjustment (e.g., cooperation; Keller et al., 2014) during task performance.

Other dimensions of skill aside from coupled action exist (e.g., knowledge of rule systems), but here we argue that action coupling is a central component that potentiates other dimensions of skill that are relevant in scenarios involving avatars. The more stably action is coupled, the higher the potential for skill. Initially, as players use a controller or other input system to manipulate the avatar in the digital environment, they are establishing associative links between behaviors (e.g., button presses, trigger pulls, or mouse clicks) and the subsequent actions they perceive the avatar performing onscreen. With continued use of the avatar, these links strengthen (i.e., become more stable).

With sufficient practice, the user’s ability to couple input actions to outcomes executed can become automatized/intuitive (i.e., entirely/very stable). To illustrate, initially as a novice player, Binta may have needed to look down at the control system to know which button to press to achieve her desired outcome. Being a novice player, Binta would accordingly be oscillating her attention from the controller to the screen to establish the link between the input and output systems. With time, Binta may become an intermediate or expert user who has practiced to the point that the input-output link is well-established as a stable cognitive association (i.e., a deep attractor). At this stage of skill development, Binta may have so thoroughly coupled her input behavior to the outcomes that her button presses no longer require attentional oscillation (Lang et al., 2018). As players focus their attention on the avatar and digital environment, rather than artifacts of the physical environment (e.g., the control system), their action coupling produces an ongoing feedback loop from the user to the digital environment through the avatar. In other words, the highly interactive context of games facilitates ongoing opportunities in which the user responds automatically and thoughtfully to information from the digital environment.

Given the connection to automaticity, action-coupling influences information processing by reducing the processing demands related to using an input system (e.g., a controller). Users have limited resources with which to process information in their environments. Humans evolved perceptual and cognitive adaptations such as action coupling that support the conservation of energy. This notion is summarized in varying areas and sometimes appears described as the concept of limited capacity processing (e.g., Lang, 2006) in which a finite pool of cognitive (and perhaps perceptual; Fisher et al., 2018) resources exist within the human body. Automatic or thoughtful allocation of those resources occurs depending on users’ motivations and the characteristics of artifacts in the informational environment. Humans’ limited capacity to process information influences what they remember *via* subprocesses of *encoding*, *storage*, and *retrieval* (Lang, 2006). Encoding is creating a mental representation of information. Storage is connecting encoded information to existing memories. Retrieval is reactivating relevant stored information. As explained by Fisher et al. (2018, p. 271), “cognitive resource limitations can inhibit the successful performance of any of [the three memory] subprocesses, reducing processing performance and influencing outcomes such as enjoyment, learning, persuasion, and many others.”

Ultimately, humans’ limited capacity to process information influences what aspects of an experience with an avatar a player attends to, how thoroughly users store information related to those experiences, and the player’s ability to later recall and ruminate on those experiences. More thorough action coupling should reduce the resources necessary to process video game events thoroughly. Thus, once a player’s action coupling has become automatized, they should have more resources available to thoroughly process experiences with avatars. In video game contexts, this means that expert players should be able to form a robust basis for the UAB compared to novice players.

### Embedding in Digital Environments Provides Experiential Spaces

The sensory presentations of digital environments sometimes mimic terrestrial (i.e., earthly) environments. The exact presentation of this information may range in visual styling from realistic simulations (e.g., the mountain environments of *The Elder Scrolls V: Skyrim*) to artistic abstractions (e.g., the cel-shaded island environments of *The Legend of Zelda: Windwaker*). Other times, digital environments depart greatly from terrestrial environments (e.g., the gravity-defying loops in *Sonic the Hedgehog*, the aquatic blitzball fields in *Final Fantasy X*). Sensory renderings (e.g., graphical display, sound) primarily serve to transmit information for perceptual intake and responsive action. In other words, digital environments present information to which users attend and respond and those responses become more stable as digital environments draw users in.

As action coupling stabilizes and a user’s attention shifts to focus on stimuli of the digital environment (i.e., the artifacts and events), they become embedded in the digital environment. Embeddedness occurs when an environment facilitates and constrains behaviors (Clark, 2008). Digital environments often present rich sensory information that can encourage feelings of being located in the environment (i.e., feeling present; Wirth et al., 2007). Embeddedness is related to concepts such as presence (i.e., the psychological sense of non-mediation in a digitally mediated environment; Lombard and Ditton, 1997); yet, embeddedness is distinct from these experiential concepts as it is premised on the behavioral input from the user and subsequent constraining of behavior by the digital environment. Indeed, feelings of presence in an environment are likely outcomes of embeddedness (Lynch, 2017).

#### Embeddedness and Nested Systems

People exist simultaneously in various environmental systems. We contend that these systems are nested based on the relative permanence of the system’s elements. Systems that have high degrees of permanence are those that people generally cannot leave and, thus, are constantly constraining and facilitating behaviors. These types of systems are *obligate* systems and are “more or less permanent, at least relative to the lifetime of their parts” (Wilson, 2002, p. 630). The physical environment, for instance, is an obligate system with elements that constrain and facilitate behavior. For instance, the physical environment of Earth has gravity, which is one of its elements. As people go about their daily lives, gravity allows them to adhere to the surface of the ground, affording behaviors such as walking. However, this same element (i.e., gravity) also constrains behaviors, as well. People generally account for gravity as they descend staircases lest they find their way to the bottom in a faster and more painful way than they had intended. Systems that have low degrees of permanence are those that people may enter and leave at will. Wilson (2002) refers to these sorts of systems as *facultative*, describing them as “temporary, organized for a particular occasion and disbanded readily” (p. 630). While embedded within a facultative system, the system constrains and facilitates behaviors, but people can abandon the bounds of the system at any moment. We contend that digital environments are examples of facultative systems.

**FIGURE 1:**
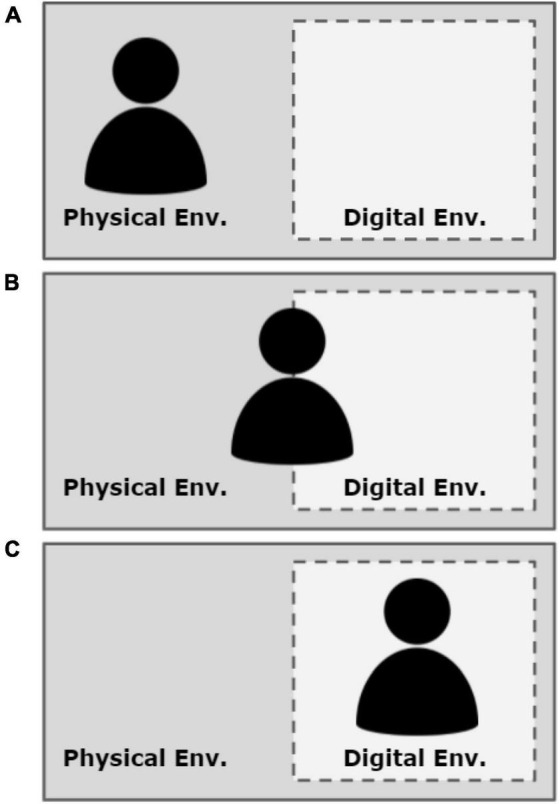
**(A–C)** Illustrating Binta’s states of embeddedness and nested physical (obligate) and digital (facultative) environmental systems. The solid bounding box of the physical (obligate) environment aims to illustrate its relative permanence/impermeability as compared to the dashed bounding box of the digital (facultative) system.

Given that digital environments are facultative systems, they are nested within obligate (and superordinate) physical environments (see Figure 1; adapted from Lynch, 2017). Although the obligate physical environment constrains/facilitates behaviors continuously, a user can still strongly attend to the facultative digital environment. Specifically, the more thoroughly and stably a user’s action is coupled, the more they are attending to ongoing events and elements of the digital environment. Put another way, the more expert a player, the more thoroughly they can become embedded in the digital environment. We note that this does not necessarily mean that they *do* become more thoroughly embedded in the digital environment, as we will consider further in this section. However, generally, as a user perceives information from the digital environment and acts in response to it, motivational systems within the user should begin to attune to the digital environment more than the physical environment. Thus, the degree of a user’s embeddedness in a digital world predicts the focus of motivational resource allocation and subsequent information processing.

#### Embeddedness and Motivational Attunement

A user’s skill can determine how much they attend to a digital environment. Accordingly, skill may predict the way that the user’s body and mind respond to the artifacts of the digital environment. Users have embodied motivational systems that orient and direct human feeling, thinking, and behaving. Specific to UABs, the artifacts and events of the digital environment can present motivationally relevant stimuli (e.g., threats, opportunities) that, in many instances, require the user-avatar to respond to them. Motivational systems automatically (i.e., preconsciously) invigorate in response to perceived stimuli in the environment. Specifically, there are two evolved motivational systems (Cacioppo et al., 1997; Cacioppo and Gardner, 1999). The appetitive system promotes opportunity seeking behaviors (e.g., finding food) and the aversive system supports threat avoidance (e.g., evading predators; Lang et al., 2000). These systems also underlie the experiences of emotions (e.g., joy and sorrow). Accordingly, motivational activity gives rise to emotional response, motivated cognition (e.g., affective states, attitudes), and subsequent behavior (Lang, 2006).

When the user is not embedded in a digital environment, characteristics of the physical environment dominate the user’s motivational system activation (Lee, 2017). Referring to Figure 1A, in this scenario if Binta receives a text message on her phone, she would likely notice it, read it, and respond to it quickly. However, as she becomes more embedded in the digital environment, characteristics of the digital environment increasingly dominate her motivational systems activity. When deeply embedded in the digital environment, characteristics of the physical environment become minimized (see Figure 1C). In other words, the digital world cognitively displaces the physical world. In this scenario, if Binta receives a text message on her phone, she may consciously ignore it or fail to recognize the notification altogether. Finally, Binta may be minimally/moderately embedded in both environments (see Figure 1B). In this scenario, if Binta receives a text message on her phone, she would likely notice it and may even read the notification while trying to maintain her control over the avatar in the digital environment.

In particular, comparing Binta’s experience illustrated in Figure 1A to her experience illustrated in Figure 1C is useful for understanding how embeddedness orders motivational system activation and subsequent information processing. Because Binta in Figure 1A is fully embedded in her physical environment, her motivational systems are oriented to seek opportunities and avoid threats in the physical environment. As a result, she processes information from, and relevant to, her physical environment (e.g., responding to hunger, responding to her phone, recognizing the states of others in the room, etc.). In contrast, Binta in Figure 1C is deeply embedded in the digital environment. Therefore, the opportunities and threats present in the digital environment orient her motivational systems and direct information processing. When fully embedded in the digital environment (i.e., Figure 1C), Binta may fail to recognize her own hunger, her phone, or the state of her physical environment (e.g., time of day). This would be quite a different experience for Binta compared to times when she is not embedded in the digital environment whatsoever (i.e., Figure 1A).

### Skill and Affordances

As a user channels motivational invigoration through coupled actions to have the avatar act in the digital environment, they are engaging in behaviors the environment facilitates. Considering affordances is one way to conceptualize the behaviors an environment potentiates for a user. *Affordances* are action potentials that emerge from the interaction of a person in an environment (Gibson, 1986). Certain features of environments afford certain behaviors (e.g., the ground enables standing; another person affords conversing) whereas other features do not afford certain behaviors (e.g., a body of liquid water restricts standing; rocks do not afford conversation; Gibson, 1986). Similarly, digital environments and avatars offer affordances to users (e.g., Eden et al., 2018; Lee et al., 2021). Because affordances emerge based on interactions between the user and the environment, variance in characteristics of the environment or the user should produce distinct affordances. Here, we will first briefly consider variance in the digital environment and then consider the ways that skill produces distinct affordances.

Digitally mediated environments structure and form human thought and behavior through their organizing elements. We consider a digital environment’s organizing elements to be sensory presentations (e.g., what the user perceives), mechanics, and rules (Meyrowitz, 1993; Strate, 2008). Mechanics are of central importance when considering affordances, as they are the coding parameters that dictate what *can* occur in a digital environment (Boyan and Banks, 2017). Mechanics also usually determine the exact way an action manifests in the digital environment for users to perceive, which contributes to the action coupling process.

Mechanics formally bound the affordances of the digital environment by limiting what actions are possible. Digitally rendered surfaces may enable standing and liquids may not, but the opposite could just as easily be true given different coding parameters (see Gaver, 1991 and Eden et al., 2018 for further consideration of simulated environments and affordances). Accordingly, users may perceive environments to afford behaviors that do not align with actual affordances of the environment (Gaver, 1991). Although this phenomenon occurs in physical environments (e.g., wax fruit does not afford eating), digital environments abound with these scenarios because they are artificially rendered. In other words, it is often the case in digital environments that simulated approximations of physical world artifacts do not afford their usual behaviors. For example, in the *Fallout* series, some doors afford opening for users to explore an indoor space; yet, many doors in this series appear to function as doors, but do not afford opening. Similarly, some environments afford unexpected outcomes. For instance, many video games feature bodies of water that afford avatars drowning rather than swimming (e.g., some games in the *Grand Theft Auto* series).

**FIGURE 2:**
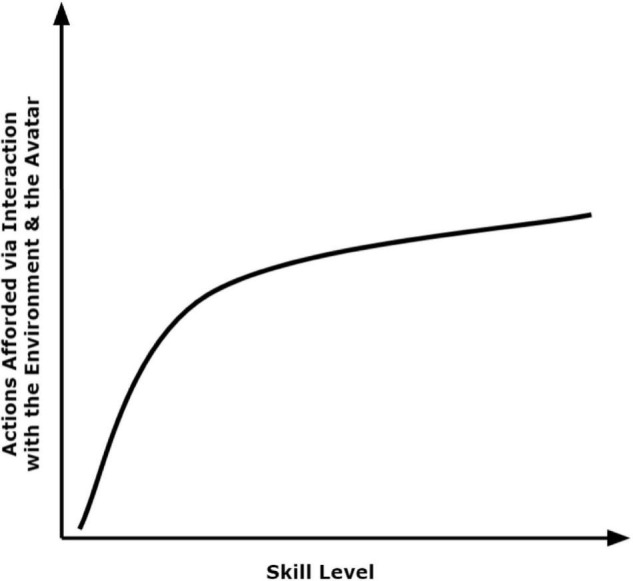
The proposed logarithmic shape characterizing the relationship between user skill and afforded behaviors.

Although environments can produce distinctions in affordances, the human centric factors that produce these distinctions are where we focus our theorizing. Skill predicts what digital environments afford to users. This is, in part, because the action coupling that underscores skill predicts distinct behaviors available to the user. The afforded actions of a digital environment and avatar likely increase rapidly (e.g., logarithmically) as skill advances from zero (i.e., complete novice) and then increases more slowly as the user transitions from moderate- to high-skill (i.e., expert; see Figure 2). In many video games, an expert user would have a broader range of actions available to execute than a novice user. Thus, skill should determine the scope (i.e., the number and type) of afforded actions in digital environments. For example, a digital environment may afford only walking straight forward to a novice user; even looking in specific directions may not be afforded to a user whose action is barely coupled from input to output. For the expert player, however, the same digital environment may afford walking, gazing accurately in 360 degrees, climbing, swimming, and more.

Returning to the intersection of a user’s skill and the characteristics of a digital environment, we now advance more theoretically compelling statements than those advanced when solely considering the environment. The organizing elements (e.g., mechanics) of some digital environments are very simple. Other environments can be incredibly complex in terms of their organizing elements. Colloquially, players and game creators often describe the relationship between skill acquisition and afforded behaviors as the learning (or skill) curve. Some games (e.g., the *Souls* series) have garnered reputations for having “steep” learning curves characterized by the high degree of challenge the environments present. Players must invest significant time and effort in playing to surmount these challenges. By contrast, some games present “shallow” learning curves (e.g., *Mario Kart*) in which players have to invest relatively less time and effort to achieve mastery over the challenges.

For instance, in the game *Morkredd*, the mechanics and rules are nearly one-dimensional. The only actions afforded are walking the avatar across the environment’s ground to progress and flipping levers. The only rule is simply to avoid the dark areas of the level to progress. Similarly, the control systems for *Morkredd* are also low in complexity; players use one joystick and one button of a controller to navigate the avatar through the lit areas of the otherwise shadowed digital environment. Given these parameters, action coupling in this game should happen quite quickly even for novice players and would seem to cascade to produce similar patterns of affordances between expert and novice players. Thus, some games have conditions in which it seems unlikely that novice and expert players’ actions and experiences would be distinct. However, research indicates that even in games with simple mechanics, rules, and control systems, novice and expert players engage in distinct action patterns based, we contend, on how skill distinguishes affordances.

Using the digital game *Tetris*, Kirsh and Maglio (1994) demonstrated how skill influences several principles of the connections between action, perception, and cognition. Specifically, Kirsh and Maglio (1994) distinguished between *pragmatic action* and *epistemic action*. Pragmatic actions are those that function to allow an individual to advance toward a goal. Epistemic actions are those that facilitate easier, faster, or more reliable computations within an individual. In *Tetris*, users must rotate and situate blocks (i.e., zoids) to completely fill rows in a playing grid. The organizing elements of *Tetris* will be familiar to many, but the point here is that the game is relatively simple. However, Kirsh and Maglio’s (1994) findings showed that expert players more frequently and more quickly engaged in extra rotations of the *Tetris* zoids compared to novice players. The extra rotations performed by experts did not directly bring the players closer to their goals. In fact, at first glance, the extraneous rotations seem to expend precious moments of time for the players, yet their proficiency in the game compared to the novice players could not be questioned.

Kirsh and Maglio (1994) describe three primary reasons why the extra rotations performed by the expert *Tetris* players allowed them to outperform the novice players. First, the rotations *reduced the computational resources* necessary for the experts to effectively determine where the zoid was in space relative to the stacked blocks at the base of the level. Second, the rotations *reduced the time* it took for expert players to identify matches between the shapes of zoids and the contours located at the level’s base. And third, the actions *increased the consistency* with which the expert players extracted information to find advantageous resolutions to the zoid and contour matching. In other words, experts leverage epistemic action to reduce computational processing and action decision costs. Therefore, the “rotate zoid” button on a controller may merely afford rotation for novice/intermediate *Tetris* players (i.e., rotate to fit). By comparison, the same button affords rotation for the expert, but also experimentation (i.e., rotate to find)—akin to shuffling Scrabble tiles rather than merely staring at the jumble of letters.

Oftentimes epistemic actions, as compared to pragmatic actions, may seem to be ineffective or adverse to goal achievement. However, Kirsh and Maglio (1994) challenged the notion that epistemic actions were, in fact, disadvantageous by comparing the actions of expert and novice players of the game. The mechanics of *Tetris* allow novice and expert players similar actions (i.e., rotations; translations, or the left-to-right horizontal shifts of zoids). However, as Kirsh and Maglio (1994) demonstrate, the extra rotations and translative actions produced different affordances for the expert players that resulted in reduced computational costs. Expert players’ actions provided new information earlier in the game than novice players and simplified the process of matching zoid and contour.

Taking the implications of Kirsh and Maglio’s (1994) findings and considering them in the context of digital environments that feature avatars, the potential for vastly different experiences emerges. We argue that their findings provide evidence that affordances of digital environments may differ based on distinctions in user skill. Given that certain affordances are unavailable to novice users, the experiences that underscore and give rise to certain types/qualities of UABs also remain unavailable to these low skill users. As skill builds, users should come to respond automatically to the digital environment and changes therein. Accordingly, a user may manipulate the avatar in certain ways to understand what is afforded by the digital environment. Take, for example, the differences in the way a player may use their avatar to learn more about the environment and actions afforded in a platformer game. Platformer games typically require a player to progress by maneuvering their avatar across uneven surfaces, often jumping, climbing, or performing other similar actions to reach distant locations. Expert players may use the avatar to learn more about the environment, for example, jumping across a pit and attempting to jump again despite the character being in midair (i.e., performing a double jump).

The expert player’s advancement is afforded by double jumping, whereas advancement might not be afforded to the novice player for whom the affordance of double jumping is unavailable. This also illustrates how skill advances with time. Even if the novice attempts to jump and the avatar dies by falling into the pit of lava below, the novice may now understand that this simple act of a single jump does not afford advancement. The novice player might initially become frustrated at having their goal impeded, experiencing aversive motivational activity and the corresponding changes to information processing. These processes should characterize the nature of the novice player’s experience with the avatar, perhaps even resulting in a strained UAB that the user may not find meaningful or enjoyable.

Yet, if the novice user persists, over time they may begin to initiate creative, epistemic actions that may not obviously advance toward the goal (e.g., returning to the level start to see if they missed something, attempting to walk rather than jump across the pit, etc.). These actions would allow them to understand what is possible in the digital environment. The expert player may use their avatar to explore the limits of what is possible by discovering new ways to act in the digital environment. The explorations may function to offload cognitive resources into the environment, as Kirsh and Maglio (1994) contend. The explorations could also reveal new ways to play that ultimately change how the player thinks about the digital environment and their avatar. Returning to Binta as an example, she may discover a glitch that allows her avatar to clip through a section of a level (i.e., walk through an object intended to be solid and untraversable), reducing her overall play time and quickening a speed run (i.e., a rapid completion of a game level). Binta may also discover novel sequences or combinations of items she can equip to her avatar to produce new affordances. For example, she might stack augmentations to create an exceptionally damage-resistant avatar. These explorations may also have complex social considerations. For example, in many massive multiplayer online games, players use avatars to engage in group-level cooperation and conflict. Users may form, maintain, or end allyships that have implications for understanding social affordances. The expert player should have experiences in the digital environment that would allow them to capitalize on the afforded actions of a given digital environment. As expert players do so, they should–by comparison to the novice–experience stronger emotional responses potentiating meaningful bonds with their avatar (e.g., feeling efficacious, powerful, etc.).

## Advancing Skill as a Predictor of User Avatar Bonds

A great deal of work exists on the nature of user-avatar interactions (see Nowak and Fox, 2018). Our focus on explicating user skill as a mechanism inherent in all users aims to provide a conceptual foundation that explains the UAB and its associated outcomes across, if not all, at least a broad range of contexts. In pursuit of our goal, we have applied a definition of an avatar to encompass all representations of the user’s agency in a digital environment. Thus, avatars may be crude or sophisticated, simple or complex, but not necessarily human, animal-like, or even in a centralized form. Our focus on the transmission of agency through the avatar provides a theoretical context in which skill produces tractable contentions (Chaffee and Berger, 1987). Below we consider how scholars can apply our conceptual definition of skill operationally and detail a few studies that have informed our theorizing in order to demonstrate skill’s influence on users’ attention and responses to stimuli that inform the development of bonds with avatars.

### Operationalizing Skill Defined as a Situated Concept

We contend that skill is most likely to be useful to scholars when operationalized idiosyncratically. Accordingly, it is relevant for scholars to understand and use specific input-output parameters when considering how skill should influence player experience. Scholars taking this approach may find it fruitful to validate skill conceptually through measurement of related psychological states such as feeling competent (e.g., Ryan et al., 2006; Lynch, 2017). As we previously considered, the same abilities and experience developed in a first-person shooter would not necessarily translate into a platformer or a role-playing game. Action coupling would occur differently (e.g., faster) in some games than others and similarly differentiate affordances. The control systems that produce user actions *via* avatars range from very low complexity (e.g., simply pressing a single button to advance a conversation) to having great complexity (e.g., navigating environments with 360 eye gaze control and movement while using items in real time). Importantly, complexity can vary dynamically over the course of a game and is often determined by decisions of users (Klimmt et al., 2009). For example, in many fighting games players can choose between selecting technical controls, which demand precise timing and inputs to control the avatar to their full potential, or simple controls, which limit the avatar’s range of function, but are easier to use.

Although considering the idiosyncrasies of digital environment input-output systems may seem overwhelming to researchers developing their study designs, as described previously, certain conventions (e.g., genre) effectively summarize types of skills reducing the breadth of how scholars might operationalize skill. As in the example of the expert who navigates the platformer game with ease, mechanics such as double-jumps are common in games of this sort, but may have no relevance outside of games in this genre. Thus, scholars may find it useful to differentiate between the exact parameters that define skill within a situated context.

For instance, Matthews and Weaver (2013) identified distinct patterns of outcomes between novice and expert players in violent video games. The authors also made these observations across two distinct video games–*Call of Duty: World at War* (COD) and *Grand Theft Auto IV* (GTA IV). Had the authors considered the distinct characteristics of the skills required to meet the challenges of these different games, they may have gleaned additional insights into the experiences of players in their study. For instance, in GTA IV, players have a broad array of tasks they can spend time with whereas in COD nearly the entire focus of the experience is perpetrating and avoiding violence. Accordingly, expert players’ experiences likely differed in other ways that would have potentiated distinct bonds to the avatar among other outcomes of interest to social scientists.

### Characterizing Relationships Between Skill, Motivation, and User-Avatar Experiences

Ultimately, users need some level of skill to accomplish what they want to do in digital environments. In some instances, this means having sufficient skill to achieve a desired goal, but this may also be more broadly achieving desired *states* (e.g., feeling competent, beautiful, competitive, etc.). To reiterate, we do not suggest that skill is simply a mechanism to outcomes such as winning or completing experiences. Instead, skill is the ordering parameter for what a game affords, facilitating the user’s ability to manipulate the avatar in such a way to produce intended states.

As we have reviewed, motivational systems fuel emotional, cognitive, and behavioral responses within the user (Cacioppo et al., 1997). These systems are constantly operational and invigorating automatically in response to the dynamic environment. Expert players may achieve desired motivational states more easily than novice players (e.g., feeling dominant at will), which in turn may potentiate stronger (or more diverse) bonds with avatars early in the development of the interaction. Although avatars themselves may have inherent motivational relevance to users (e.g., an avatar designed to look and act like a puppy may have appetitive motivational relevance because it is cute and friendly), the more interesting point in our view is that the user’s skill should shape their motivational response to the avatar. Furthermore, the nature of the user’s motivational response should change as the user’s skill changes. To illustrate how skill helps users achieve desired states, below we detail the nature of a specific pattern of motivational system activation and how user skill influences the pattern.

As explained, general principles of motivational system activation apply readily to explaining UABs. Generally, motivational systems automatically allocate resources toward processing certain types of information (e.g., threats) in a given context. For example, more proximate stimuli typically evoke stronger motivational system activation and especially so if the stimuli are dangerous, which would lead to greater consumption of cognitive resources. Motivational systems respond automatically to stimuli with distinct patterns of activity emerging over time in the appetitive and aversive systems, respectively. Positivity offset and negativity bias (i.e., PONB) is among the most notable of these activation patterns. *Positivity offset* describes the subtle invigoration of the appetitive system that occurs when in a resting state (i.e., at low levels of arousal; Cacioppo et al., 1997). Low-level appetitive activation promotes opportunity-seeking behaviors. This subtle activation does not occur in the aversive system in a resting state. Instead, a *negativity bias* characterizes activation of the aversive system. Negative stimuli invigorate the aversive system powerfully and rapidly, producing a bias in processing where aversive activation draws greater levels of resources in a shorter span of time compared to appetitive activation (Cacioppo et al., 1997). Take, for example, a scenario in which a hiker encounters a bear. This scenario would likely elicit a strong aversive response (i.e., fear) in the hiker. On the other hand, a hiker who is resting by a stream and sees a butterfly fluttering above the water would likely experience minimal invigoration of appetitive systems (i.e., contentment).

Despite their immateriality, digital environments encourage ongoing attention and subsequent behavioral enactment. Human perceptual and motivational systems do not immediately distinguish digitally mediated stimuli from those physically in the environment (Reeves and Nass, 1996). People then act in motivated cognitive states to either approach/avoid different things in the environment. Functionally, motivational activation serves to promote cognition and adaptive behavior, but how those outcomes manifest depends on characteristics of the person in a given environment (Buck, 2014). For instance, the strong invigoration of aversive systems in response to a proximate threat promotes fight *or* flight behavior (Lang et al., 2000). Skill may very well differentiate whether a person fights or flees in the face of a threat (e.g., Lang et al., 2018). An expert may have the ability to engage in defensive and offensive maneuvering whereas a novice may only have escape behaviors available to them. Furthermore, as users’ skills develop, they likely become increasingly capable of manipulating avatars to achieve desired motivational states intentionally (e.g., coordinating social events in games among a team of players to satisfy a need for relatedness). This possibility is poignant, as a user’s ability to create an intended motivational state in themselves opens experiential opportunities.

#### Summarizing Short- and Longer-Term Outcomes for User-Avatar Bonds

Because user skill helps determine the likelihood of motivational states, skill should influence the way the user manipulates the avatar in response to motivationally relevant stimuli. To elaborate, an avatar’s current state can result in fleeting motivationally relevant states for a user (e.g., the avatar is in danger of taking damage). Motivational responses to that stimulus should invigorate relatively universally, but skill would predict the nature of how the user’s agency flows through the avatar (i.e., how a user-avatar responds to the stimulus). For example, imagine Binta is guiding her avatar’s descent down a snowy slope during a skiing event and capturing flags as she passes check points. Binta’s appetitive motivational system should invigorate and perhaps more so as she embeds deeply in the environment and nears the end of the event. In the short-term Binta may feel excitement and use the subsequent motivational invigoration (i.e., appetitive systems activity) to have the avatar respond to challenges in the environment. If Binta were an expert player in this scenario, she may perform jumps and tricks to gain advantages in the game that would speed up her descent and improve her overall performance. If Binta were a novice player, she might simply attempt to navigate without crashing her avatar into trees.

Some recent empirical evidence supports this theorizing. For example, Lang et al. (2018) investigated how experience in a digital environment differentiated people’s patterns of behaviors and afforded outcomes. Specifically, they content analyzed people’s driving behaviors in *Grand Theft Auto IV* (GTA IV). They found that novice players performed less risky behaviors while driving and, as a result, hit fewer objects and people while playing the game than expert players. Although this outcome seems counterintuitive, the authors posited that expert players took greater risks and caused more mayhem because they understood that such behaviors could confer benefits (e.g., enjoyment, in-game resources, etc.) with minimal in-game losses. Novice players, on the other hand, drove in ways that mirrored driving in the physical environment (e.g., driving slow, not hitting cars, etc.). In other words, the skill possessed by expert players facilitated patterns of behavior among them that were more consistent with the digital environment of GTA IV than the physical environment.

Similarly, Lynch (2017) investigated whether a user’s skill influenced their emotional reactions when being hunted by a monster in a digital environment. The avatar in the selected game possessed no offensive abilities. As a result, players had to hide or otherwise engage in avoidance behaviors for their avatar to survive the experience. A central independent variable in this study was the size of the avatar. Those who used a small and slender avatar reported less fear when playing the game compared to people playing as a large and muscular avatar. Similar to the previous example, this outcome seems counter intuitive. However, this effect only emerged for expert players. In interpreting this finding, Lynch suggested that higher skilled players may have recognized that a smaller avatar afforded better threat avoidance than a larger avatar. By comparison, novice players reported no differences in fear across condition. Notably, however, novice players reported less fear overall than expert players lending support to the notion of motivational attunement when embedded. Other recent work demonstrates that the principles of motivational systems and subsequent behavioral enactment do indeed emerge reliably in highly interactive digital environments (Lee et al., 2021).

The relationship between skill and fostering specific motivational states also underlies more persistent, longer-term feelings about avatars (i.e., attitudes about or feelings toward one’s avatar). Returning again to Binta, she may customize her avatar’s appearance with the motivation to achieve an esthetically attractive form. This action would require a base level of skill to navigate the digital environment and successfully manipulate the avatar to afford appearance customization. If Binta is pleased with the appearance of her avatar, then her appetitive systems may invigorate at low levels any time she interacts with the avatar. This could produce positive attitudes in Binta toward her avatar such that she reports liking the avatar. This generally positive orientation may also then influence the way that Binta uses her avatar. For example, Binta may avoid engaging in activities that would temporarily mar the avatar’s appearance because she is motivated to maintain the avatar’s esthetics. Likewise, she may engage in epistemic actions that seemingly undermine goal attainment in pursuit of motivational states distinct to her as an expert player. For instance, she might choose esthetically pleasing equipment at the expense of power or capability (e.g., a suitcoat instead of armor). Her skill, however, would allow her to capitalize on a range of affordances that would effectively recoup on an otherwise reduced advantage.

As another hypothetical example, if Binta has used her avatar to explore tragic narrative themes over the course of the game experience, she may develop feelings of sadness or pity with respect to her avatar. The avatar would thus have negative (i.e., aversive) motivational relevance for Binta based on her experiences using the avatar. Accordingly, Binta may feel motivated to finish extra narrative or exploratory content to achieve a positive rather than negative ending to the avatar’s narrative. This hypothetical trajectory illustrates a player experience that would offer an opportunity for social scientists to learn more about the influence of skill on long term patterns in player behavior.

#### Information Processing and Experiential Outcomes Distinguished by Skill

Notable work does exist examining information processing with respect to avatars (e.g., Ganesh et al., 2012; Ratan and Dawson, 2016; Taylor and Dando, 2018). Yet, memory processes are central factors in understanding characteristics of UABs that remain underexamined by researchers. Because skill influences motivational attunement and activation, we contend that skill would likewise predict the processing of information by reducing processing demand and influencing outcomes such as working memory (e.g., Read et al., 2018).

Accounting for motivation and information processing provides a framework for researchers to classify and predict outcomes of interacting with an avatar in a digital environment. For example, the motivational tenants of positivity offset and negativity bias predict what content is likely to demand the user’s attention (Cacioppo et al., 1997). For example, Reeves and Nass (1996) explain how aversive content commands attention and influences the storage of information predictably. People tend to store details *immediately before* the onset of an aversive stimulus poorly (i.e., retroactive interference). In contrast, people tend to store details *following* the onset of an aversive stimulus well (i.e., proactive enhancement). Yet, skill may predict distinctions in processing this information because it may differentiate the amount of resources dedicated to aversive systems’ activity in this moment. Generally, if Binta was guiding her avatar through a complex maze to reach a treasure chest and a horde of monsters attacked as she reached the room with the chest (i.e., an aversive event), her memory of the maze—especially the portion encountered immediately prior to the aversive event—would be poor. By comparison, Binta would better remember the route she took when escaping the hoard (i.e., details *after* the aversive event).

These divergent outcomes in memory occur because strong aversive reactions motivate people to encode what is happening in the moment at the cost of reduced storage and retrieval. However, skill should influence such well documented patterns of memory construction predictably. For example, a skillful player may resist negativity’s influence on processing due to their ability to anticipate conflict and react accordingly. Principles such as this abound in the literature on motivational systems. We contend that they present opportunities to specify how users at differing levels of skill interact with the motivationally relevant characteristics of the avatar and the digital environment to specify what parts of the UAB users find memorable.

Finally, skill should help predict broader experiential outcomes by considering the relationship between embeddedness in systems and motivational attunement. Researchers have studied experiential phenomena with respect to how immersive technologies can facilitate states that dominate one’s senses (e.g., pain management *via* virtual reality technologies; Pourmand et al., 2018) and have used a variety of measurement techniques to observe the allocation of resources during psychologically immersive states (e.g., secondary task reaction time; Keene and Lang, 2016). However, less attention has been given to ways in which skill predicts how resources are expended as users’ embeddedness oscillates between the physical environment and the digital environment in video games. For example, in cooperative games, it is common for skillful users to engage in complex strategizing and coordination through verbal conversation with their partner while maintaining focused attention in the digital environment. The users are oscillating between perceiving and acting in the digital environment while communicating with others. These individuals may be co-located in their physical environment or located remotely, which likely matters in ways related to motivation and information processing. The extent to which the avatar draws additionally on users’ resources likely also oscillates during such interactions, but this provides just one example of how social scientists might explore how skill predicts embeddedness and its subsequent outcomes. Work on attention and information processing during media multitasking (e.g., Wang et al., 2015) would likely serve as excellent connecting points to advance knowledge on these phenomena in contexts with avatars.

### Experiences With Avatars Produce Distinct Relationships

Existing research describes how the highly interactive nature of video games and similar technologies facilitate different types of experiences relative to other types of media (e.g., film). For example, Klimmt et al. (2009) describe the experience of monadic identification with an avatar in which the player adopts and merges characteristics of the avatar temporarily into their own self-perception. Similar to our thinking, Klimmt et al. (2009) elaborate that the central mechanism driving this phenomenon is the interactive control the player holds over the avatar and that the link established through this interactive connection facilitates the psychological merger of the avatar and player. We contend that skill is a crucial, human centric concept that is, as yet, under considered in this literature.

However, working from perspectives that advance concepts of action coupling, embeddedness, and affordances, we suggest that monadic identification and similar constructs already identified in the literature of user-avatar interactions (e.g., embodiment) may present excellent junctions in the avatar literature to advance our understanding of how skill predicts what avatars afford users in digital environments. Specifically, we suggest that avatars may afford *cognitive extension* into the digital environment for expert players. Cognitive extension is when people control or otherwise exert power over an object and the object itself becomes part of the cognitive system. Cognitive extension exists within a broader construct that conceptualizes cognition as embodied, embedded, and extended (see Clark, 2008). Communication researchers (e.g., Clayton et al., 2015; Bailey et al., 2021) ground cognitive extension in perspectives such as Gibson’s articulations of affordances and perception-action linkage (Gibson, 1986), Belk’s extended-self theory (1988), and Clark’s notion of the negotiable body (2008).

The components that define the broader construct of cognitive extension are robust, complex, and paradigm-defining. With time and effort, people can come to view an object as part of themselves (McClelland, 1951; Belk, 1988; Clayton et al., 2015; Bailey et al., 2021). For instance, smartphone users describe viewing the device as an extension of themselves that not only enhances their physical or intellectual capabilities (i.e., functional extensions), but one that has user-like characteristics (i.e., anthropomorphic extensions) and becomes difficult to uncouple from themselves (i.e., ontological extensions; Park and Kaye, 2019). However, it is not simply the coupling of the object and the person that produces cognitive extension. Rather, the object–in our case, the avatar–becomes part of the person’s cognitive system, facilitating a new way of thinking and perceiving the environment (e.g., *via* epistemic action). We assert that this presents a provocative direction for considering how skill determines user-avatar interactions and one, to our knowledge, that scholars have not formally considered. Future work could do more to advance these ideas in compelling ways.

## Conclusion

Summarizing the relevance of skill in these processes, expert players’ action input should be stably and deeply coupled to perceived outcomes in the digital environment. Their attention should be strongly attuned to the digital environment, potentiating motivational invigoration to stimuli in the digital environment. Accordingly, they would typically recognize challenges and opportunities in the digital environment quickly. The relative strength of their action coupling should reduce the processing demands of input, leaving plentiful processing resources for encoding, storing, and retrieving information from the digital environment. And, finally, the diminished effort in coupling input to perceived outcomes facilitates more masterful expressions of the user’s agency and greater ability to meet or overcome the challenges a digital environment may present (e.g., solving puzzles).

For expert players, then, the experiences they have with an avatar when embedded in a digital environment should seem proximate, invigorating, and consequential. For novice players, the experiences they have with their avatar may feel less robust and inconsequential. The expert and the novice player likely experience qualitatively distinct UABs even if the avatar is fundamentally the same. If the bond that a user has with their avatar is formed by their experiences together, then the nature of what the user can do in the digital environment is consequential.

## Author Contributions

TL, NM, and MG contributed to the conception of the framework. TL and NM wrote the first draft of the manuscript. All authors contributed to manuscript revision, read, and approved the submitted version.

## Conflict of Interest

The authors declare that the research was conducted in the absence of any commercial or financial relationships that could be construed as a potential conflict of interest.

## Publisher’s Note

All claims expressed in this article are solely those of the authors and do not necessarily represent those of their affiliated organizations, or those of the publisher, the editors and the reviewers. Any product that may be evaluated in this article, or claim that may be made by its manufacturer, is not guaranteed or endorsed by the publisher.
